# Elevated Hepcidin Is Part of a Complex Relation That Links Mortality with Iron Homeostasis and Anemia in Men and Women with HIV Infection^1^,^2^,^3^

**DOI:** 10.3945/jn.114.203158

**Published:** 2015-04-22

**Authors:** Peter A Minchella, Andrew E Armitage, Bakary Darboe, Momodou W Jallow, Hal Drakesmith, Assan Jaye, Andrew M Prentice, Joann M McDermid

**Affiliations:** 4 Department of Nutritional Sciences, Cornell University, Ithaca, NY;; 5 Weatherall Institute of Molecular Medicine, John Radcliffe Hospital, University of Oxford, Oxford, United Kingdom;; 6 Medical Research Council Unit (UK), Fajara, The Gambia;; 7 International Nutrition Group, Department of Nutrition and Public Health Intervention Unit, London School of Hygiene and Tropical Medicine, London, United Kingdom; and; 8 Division of Infectious Diseases & International Health, Department of Medicine, School of Medicine, University of Virginia, Charlottesville, VA

**Keywords:** Africa, cohort, ferritin, hemoglobin, HIV-2, inflammation, nutrition, survival, transferrin receptor, transferrin

## Abstract

**Background:** Early and chronic inflammation is a hallmark of HIV infection, and inflammation is known to increase hepcidin expression. Consequently, hepcidin may be a key determinant of the iron homeostasis and anemia associated with poorer HIV prognoses.

**Objective:** The objective of this study was to understand how hepcidin is related to anemia, iron homeostasis, and inflammation at HIV diagnosis and to investigate associations between hepcidin and all-cause mortality in HIV infection.

**Methods:** In a retrospective cohort, baseline plasma hepcidin was measured by competitive enzyme immunoassay within 3 mo of HIV diagnosis in 196 antiretroviral-naive Gambians. Iron homeostasis [hemoglobin, plasma transferrin, ferritin, iron, soluble transferrin receptor (sTfR)] and inflammation [α_1_-antichymotrypsin (ACT)] from the same plasma sample were available, as were absolute CD4 cell counts, age, gender, body mass index (BMI), and HIV type.

**Results:** Anemia was common across the spectrum of immunosuppression [CD4 cell counts (prevalence of anemia): >500 cells/μL (68%), 200–500 cells/μL (73%), and <200 cells/μL (89%); *P* = 0.032] and in men (81%) and women (76%). Increasing hepcidin was associated with iron homeostasis biomarkers (higher ferritin and lower transferrin, hemoglobin, and sTfR), inflammation (higher ACT), and key health indicators (lower CD4 or BMI, advancing age, and male gender; *P* < 0.001 except for hemoglobin, *P* = 0.021). Elevated hepcidin was associated with greater all-cause mortality in a dose-dependent manner [intermediate vs. lowest tertile: unadjusted HR (95% CI), 1.95 (1.22, 3.10); upper vs. lowest tertile: 3.02 (1.91, 4.78)]. Principal components analysis identified 2 patterns composed of hepcidin-ferritin-transferrin, with or without ACT, and iron-sTfR-hemoglobin that may distinguish inflammation and erythropoiesis iron functions.

**Conclusions:** Elevated hepcidin is independently associated with greater mortality in men and women with HIV infection, and hepcidin is also part of a complex relation linking iron homeostasis, anemia, and HIV. Understanding the mechanisms and role of hepcidin modulation may further guide evidence-based interventions needed to counter detrimental iron homeostasis and anemia in HIV infection.

## Introduction

Anemia and abnormal iron distribution are associated with increased morbidity and mortality in HIV infection (1–5). Nutritional causes of anemia include iron, folate, and vitamin B-12 deficiencies, which are considerable in regions affected by poverty (6, 7). People living in these regions are frequently burdened by infectious diseases, and the associated chronic immune activation and inflammation they experience fuel anemia of inflammation (AI)^9^ (8). Because iron homeostasis is affected by immunologic, infectious, clinical, and nutritional contributors, it acts as a barometer of overall health status.

Early and chronic immune activation and inflammation are hallmarks of HIV infection. Under inflammatory conditions, dietary iron is blocked from enterocyte release, whereas circulating iron is redistributed into cellular storage locations that include macrophages. This affects iron delivery needed for erythropoiesis, and additional HIV-associated consequences, including myelosuppression, impaired erythropoietin production, and opportunistic infections, further contribute to anemia in HIV infection (9, 10). These processes may explain why anemia is such a common hematologic disorder before antiretroviral therapy (ART) is initiated (11). Although ART reduces the prevalence of anemia for many people, a considerable proportion experience unresolved anemia or develop anemia after ART initiation (**Supplemental Table 1**).

Hepcidin is a protein integrally involved in iron homeostasis and anemia through its interaction with the only known vertebrate cellular iron exporter, ferroportin [reviewed in (12)]. Inflammation or excess iron stores lead to upregulation of hepcidin expression and synthesis, whereas erythropoiesis and hypoxia are negative regulators (13). Hepcidin inhibits iron efflux from duodenal enterocytes and increases intracellular iron retention in iron-recycling macrophages. Recent reports indicate that hepcidin may influence multiple HIV outcomes because higher hepcidin concentrations were associated with increased in vitro HIV-1 transcription (14), as well as with greater immunosuppression and tuberculosis among HIV-positive Indonesians (15). These outcomes were previously linked with other iron-related biomarkers (16–18), suggesting that hepcidin is just one contributor to the complex iron biology in HIV infection. Despite the importance of hepcidin and the iron regulation associated with anemia, and the link between hepcidin and inflammation that was shown other clinical conditions (19–21), the role of hepcidin in the context of HIV infection and HIV-related anemia remains undetermined. Consequently, this study was designed to characterize hepcidin concentrations at HIV diagnosis in relation to anemia, iron homeostasis, and inflammation and to investigate hepcidin as an independent risk factor for mortality in HIV infection in a region where both infectious and nutritional contributors are common.

## Methods

### 

#### Study setting.

Participants were recruited from a larger prospective cohort study (22) and a substudy (16, 23) in HIV-positive Gambians. Eligibility criteria included age ≥18 y and an existing baseline plasma aliquot archived within 3 mo of first HIV diagnosis. Cohort participants were provided free clinical care at the Medical Research Council Unit in The Gambia in accordance with national HIV guidelines in effect at the time of participation, including cotrimoxazole prophylaxis, measurement of immunosuppression according to absolute CD4 cell counts (FACScan; Becton-Dickinson), and symptom management. Viral load analysis was unavailable for routine monitoring at the time of sample collection. Ethical approval for this study was granted by the Joint Gambian Government/Medical Research Council Ethical Review Committee, The London School of Hygiene and Tropical Medicine, and Cornell University.

#### Follow-up and all-cause mortality ascertainment.

Participants (*n* = 196) enrolled between 5 August 1992 and 22 August 2001, with follow-up observation continuing until 1 June 2002. All-cause mortality was ascertained at study clinic visits scheduled every 3 mo. Participants who missed appointments were considered lost to follow-up if their mortality status could not be determined by medical records or at home visits. Participants were censored on the last date they were known with certainty to be alive, defined as the last date of clinic attendance or the last date seen by a fieldworker or the end of the study observation period. As is common in this region of Africa, cause-specific mortality was unavailable because autopsies, including verbal autopsies (24), were not routine.

#### Hepcidin, anemia, iron homeostasis, and inflammation.

All assays were performed by using a single heparinized plasma aliquot that was stored at −80°C. This single plasma sample was thawed and then subdivided into multiple microvials and refrozen at −80°C. A single microvial was obtained from the freezer and thawed for a second time until batch processing of each analyte. The period between refreezing and hepcidin analysis was longer than for other analytes. Although the long-term stability of hepcidin is unknown due to the relatively recent commercial availability of this assay, the presence of 4 disulfide bonds in the folded structure suggests that the peptide should maintain long-term stability (25). Assay quality controls provided by the assay manufacturer were used and performed within manufacturer-defined limits, and pooled plasma obtained from a local blood bank was used to assess quality assurance. Hepcidin concentrations were measured in duplicate by using a competitive enzyme immunoassay (Bachem) according to the manufacturer’s protocol (26, 27). Samples were diluted on the basis of known ferritin concentrations in supplied standard diluent (peptide-cleared human serum) and analyzed by using a 9-point, 2-fold serial dilution (maximum concentration: 25 μg/L) standard curve. Hepcidin concentrations were interpolated from 4-parameter logistic standard curves generated by Readerfit (www.readerfit.com). The lower limit of detection of 0.40 μg/L was interpolated at 3 SDs from the all-plate mean (e.g., wells containing diluent in lieu of hepcidin standard or sample). The 11 undiluted samples with limits of detection <0.40 μg/L were imputed with a value of limit of detection/2. Samples with concentrations outside the linear region of the curve, or those with an intra-assay CV >15%, were reanalyzed with the use of appropriate dilutions.

Soluble transferrin receptor (sTfR; R&D Systems) and ferritin (Immuno-biological Laboratories) were measured by ELISA, and limit of detections were <3 and >80 nmol/L and <2.5 and >1000 μg/L, respectively; samples with values outside of these ranges were imputed with the limit of detection value. Plasma iron was assessed by using an endpoint assay (ABX Diagnostics) and transferrin was assessed by turbidimetry (ABX Diagnostics). Hemoglobin concentrations were measured with a hematology analyzer by using routine procedures in the clinical laboratory (Coulter MD II; Coulter Corporation) and values obtained from study databases. α_1_-Antichymotrypsin (ACT), as an indicator of inflammation, was measured by using a nephelometric assay (DakoCytomation).

#### Statistical analysis.

STATA/MP 11.1 (StataCorp) was used for statistical analyses. Values in the text are presented as means ± SDs or as medians (IQRs); *P* values <0.05 were considered significant for statistical tests. Data transformation included categorization of hemoglobin, ferritin, sTfR, and iron according to clinical reference ranges. Inspection of raw data suggested that the use of only the clinical reference range would obscure meaningful transferrin associations, and transferrin was reported according to both clinical reference limits and tertiles. In the absence of an established clinical reference range for hepcidin, hepcidin was classified into tertiles. ACT is primarily a research analyte, and a clinical reference range was not provided by the manufacturer or recommended by others (28); however, for this statistical analysis, a frequently cited cutoff range was used (29).

Scatterplots and boxplots were used to graphically present bivariate associations between hepcidin and hemoglobin, iron homeostasis, and inflammation biomarkers, as well as the a priori–considered potential covariates (BMI, age, absolute CD4 cell count, gender, HIV type). Pearson correlation coefficients, Wilcoxon rank sum, ANOVA, Student’s *t *test, Spearman rank correlation coefficient, or chi-square test were used to test these associations. A Kaplan-Meier survival curve was used to graphically represent the probability of survival from HIV diagnosis according to hepcidin tertiles and compared by using the log-rank test. Univariable and multivariable Cox regression models with all-cause mortality as the main outcome were analyzed.

Principal components analysis (PCA) was chosen as a variable reduction technique given the potential redundancy (e.g., correlation) in the measured iron homeostasis (transferrin, ferritin, iron, hemoglobin, sTfR) and inflammatory (ACT) variables. Essentially, the goal of PCA was to reduce the number of observed variables to allow concentration on only those that principally contribute to variation in a meaningful way. This was done by statistically identifying groups of variables that “travel together” in components (e.g., patterns), while disregarding those variables that only contribute in a negligible way. Identified patterns represent distinct groups that are uncorrelated from other patterns. Decisions regarding which components were meaningful to retain was based on the a priori criterion of eigenvalue-1 (patterns with eigenvalues >1 were considered to contribute principally).

PCA was conducted with iron and inflammatory variables categorized in tertiles and also as continuous variables; because similar results were obtained, only tertile models are reported. After identifying principal patterns of iron and inflammation groupings, principal component (PC) scores were calculated for each individual. These scores represent a linear algebraic equation accounting for the weighting of each observed variable. PC scores were modeled as a continuous variable and analyzed in unadjusted and adjusted Cox regression. An iron homeostasis index was also calculated for each participant on the basis of the PCA-identified pattern groupings. The iron homeostasis index conceptually represents the magnitude of iron deviation and combined impact on mortality. It was calculated by using the observed concentrations measured for each iron and inflammatory variables classified into tertiles. A value of 1 was assigned to the lowest HR associated with each biomarker. In these data, the lowest HRs were associated with the lowest tertile of hepcidin, ferritin, sTfR, and ACT and the highest tertiles of transferrin, iron, and hemoglobin. All intermediate tertiles were assigned a value of 2; and all remaining tertiles were assigned a value of 3 (e.g., those associated with the greatest HRs). Values were summed for each individual, providing an overall iron homeostasis index specific for each individual and each PCA-identified pattern. The iron homeostasis index was modeled as a single continuous variable and analyzed by using unadjusted and adjusted Cox regression.

## Results

### 

#### Subjects.

The characteristics of the 196 cohort participants are summarized in **Table 1**. During the 10-y cohort follow-up period, 64% of participants died (*n* = 125), 19% (*n* = 37) were censored at the last date with known certainty to be alive, and 17% (*n* = 34) were alive at study closure. Participants censored before death or study closure were more likely to have greater absolute CD4 cell counts such as commonly observed in resource-restricted regions without universal ART access (31) and, in this study, were also more likely to be younger and female.

**TABLE 1:** Characteristics of cohort participants at HIV diagnosis^1^

	Overall (*n* = 196)	Anemia (*n* = 110)	No anemia (*n* = 30)	*P*^2^
Cohort person-years	1.8 (0.6, 4.1)	1.2 (0.5, 3.3)	4.1 (1.7, 7.9)	<0.001
Age, y	34.3 ± 9.8	33.0 ± 8.8	34.6 ± 10.1	0.42
Female, %	55	53	60	0.48
HIV status, %				0.65
HIV-1	60	65	60	
HIV-2	39	35	40	
HIV-dual^3^	1	0	0	
Absolute CD4, cells/μL (*n* = 182)	250 (92, 503)	233 (77, 498)	408 (243, 699)	0.003
BMI, kg/m^2^ (*n* = 178)	19.7 ± 4.0	19.2 ± 3.5	21.6 ± 5.1	0.005
BMI <18.5 kg/m^2^, %	37	33	33	
α_1_-Antichymotrypsin, g/L	0.41 (0.31, 0.58)	0.43 (0.39, 0.46)	0.32 (0.28, 0.36)	0.003
Hepcidin, μg/L	22.1 (3.3, 85.9)	32.2 (2.2, 88.7)	6.8 (2.1, 53.3)	0.06
Minimum/maximum	0.2/402.2	0.2/598.4	0.2/235.1	
Ferritin, μg/L	161 (37, 534)	158 (36, 643)	90 (28,191)	0.09
Minimum/maximum	2.5/1001	2.5/1001	2.5/1001	
Transferrin, g/L	1.80 ± 0.64	1.75 ± 0.67	2.00 ± 0.50	0.06
Minimum/maximum	0.21/3.15	0.21/3.36	1.26/3.15	
Iron, μmol/L	9.0 (6.4, 14.4)	8.5 (6.4, 12.6)	15.0 (10.0, 18.8)	<0.001
Minimum/maximum	1.1/41.8	3.0/41.8	3.8/31.7	
sTfR, nmol/L	24.4 (17.0, 35.5)	25.3 (21.5, 28.2)	19.8 (16.6, 23.0)	0.02
Minimum/maximum	5.7/81.0	5.7/81.0	6.3/62.1	
sTfR/log_10_ ferritin	11.1 (7.1, 19.0)	11.3 (7.3, 18.7)	10.4 (6.5, 24.1)	0.55
Minimum/maximum	2.0/203.5	2.0/203.5	2.4/102.9	
Hemoglobin, g/L (*n* = 140)	10.5 ± 2.3	9.6 ± 1.7	13.8 ± 1.1	<0.001
Minimum/maximum	5.3/16.1	5.3/12.9	12/16.1	

1 For normally distributed continuous variables, values are presented as means ± SDs; nonnormally distributed continuous variables are presented as medians (IQRs) and lower/upper minimum/maximum limits; categorical variables are presented as frequencies (%). All assays were performed with the use of plasma. Anemia was defined according to the WHO definition: men, hemoglobin <13 g/dL; women, hemoglobin <12 g/dL (30). Hemoglobin values were available for 140 participants; therefore, the sum of participants with “anemia” and “no anemia” was equal to 140. sTfR, soluble transferrin receptor.

2 *P *values were calculated by comparing group means (Student’s *t* test for normal distribution, continuous, and Wilcoxon’s rank-sum test for nonnormal distribution, continuous) and or frequencies (chi-square test) between anemic and nonanemic groups.

3 Given the small number of HIV dual diagnoses, HIV-2 was combined with the HIV-1 group for subsequent analyses.

#### Hepcidin and association with iron homeostasis, anemia, and inflammation in HIV.

Hepcidin was positively correlated with ferritin and ACT and inversely correlated with hemoglobin, transferrin, and sTfR but not plasma iron (**Supplemental Figure 1**). Higher hepcidin concentrations were correlated with lower absolute CD4 cell counts or BMI and advancing age, but there was no apparent association with HIV type (**Supplemental Figure 2**). Although men [52.4 (16.7, 96.9) μg/L] had higher hepcidin concentrations than women [11.9 (2.4, 58.6) μg/L] (*P* < 0.001), men also experienced greater immunosuppression at HIV diagnosis [CD4 cell count in men: 191 (136, 255) cells/μL; CD4 cell count in women: 292 (218, 399) cells/μL; *P* < 0.001].

Table 1 summarizes participant characteristics according to anemia classification. Anemia was associated with a lower mean BMI and more advanced immunosuppression, as expected, but it was also common across the spectrum of immunosuppression. Prevalences of anemia were 81% in men, 76% in women, and 68%, 73%, and 89% in participants with CD4 cell counts of >500, 200–500, and <200 cells/μL, respectively. Anemia was associated with a greater degree of inflammation as indicated by greater ACT concentrations (anemia vs. no anemia: 0.43 vs. 0.32 g/L; *P* = 0.003), as well as an inflammation-induced iron redistribution profile typified by comparatively greater hepcidin and ferritin and lower transferrin and iron concentrations (*P* < 0.09). Although sTfR concentrations were higher in the anemic group, suggesting the presence of iron deficiency or coexisting iron deficiency plus AI, the sTfR:log_10_ ferritin ratio was not significantly different between those with or without anemia.

#### Hepcidin and mortality in HIV.

**FIGURE 1:**
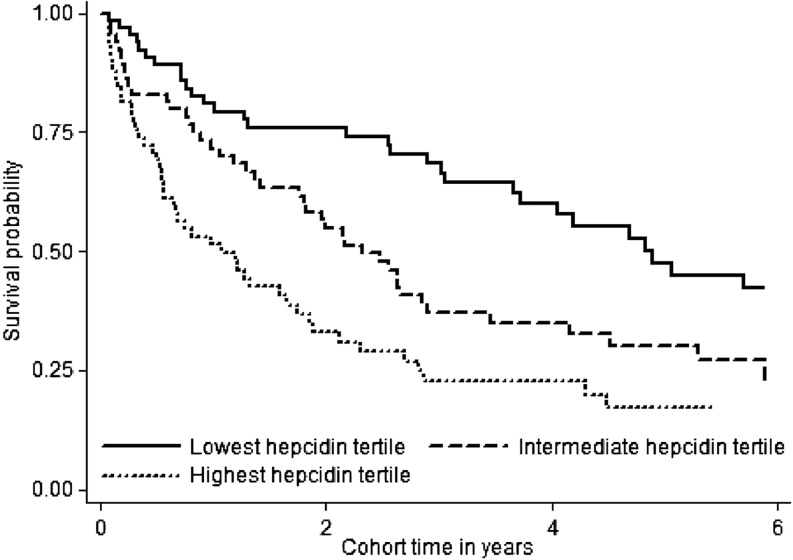
Kaplan-Meier survival curves according to hepcidin tertiles in men and women at HIV diagnosis. Lowest hepcidin tertile: ≤7.8 μg/L (*n* = 65; 33%); intermediate tertile: >7.8 to <57.6 μg/L (*n* = 66; 33%); highest tertile: ≥57.6 μg/L (*n* = 65; 33%).

**Figure 1** shows a dose-response relation between greater hepcidin at HIV diagnosis and increasing probability of all-cause mortality. This relation is further supported in unadjusted Cox regression analysis; however, the adjusted association was attenuated and not statistically significant (**Table 2**). Like hepcidin, all other iron homeostasis and inflammatory associations were attenuated in regression models adjusting for HIV type, age, gender, BMI, and immunosuppression at HIV diagnosis, with or without additional adjustment for ACT. Anemia, very elevated ferritin, and the lowest transferrin tertile remained statistically significant in adjusted analyses.

**TABLE 2:** Cox regression models of hepcidin, iron homeostasis, and inflammation in men and women at HIV diagnosis and associations with all-cause mortality^1^

			HR (95% CI)^3^		
Plasma biomarker and tertile at HIV diagnosis	*n* (%)	Clinical cutoff or tertile limits^2^	Univariate model	Adjusted model^4^	Adjusted model plus ACT^5^
Hepcidin, μg/L					
Lowest	65 (33)	≤7.8	Reference	Reference	Reference
Intermediate	66 (33)	>7.8 to <57.6	1.95 (1.22, 3.10)	0.96 (0.56, 1.63)	0.97 (0.56, 1.67)
Highest	65 (33)	≥57.6	3.02 (1.91, 4.78)	1.07 (0.61, 1.87)	1.11 (0.59, 2.08)
Ferritin, μg/L					
Lower than normal	23 (12)	<12	0.62 (0.31, 1.21)	0.61 (0.29, 1.25)	0.58 (0.28, 1.21)
Normal	98 (50)	—^6^	Reference	Reference	Reference
Elevated	45 (23)	—^6^	2.10 (1.37, 3.23)	1.57 (0.97, 2.51)	1.90 (1.14, 3.18)
Very elevated	30 (15)	>1000	3.91 (2.44, 6.28)	2.09 (1.19, 3.67)	2.78 (1.49, 5.17)
Transferrin, g/L					
Highest	65 (33)	≥1.89	Reference	Reference	Reference
Intermediate	66 (33)	>1.47 to <1.89	2.42 (1.37, 4.24)	0.78 (0.39, 1.57)	0.79 (0.40, 1.58)
Lowest	65 (33)	≤1.47	4.36 (2.85, 6.66)	1.92 (1.12, 3.31)	2.13 (1.21, 3.75)
Lower than normal	133 (68)	<2.0	2.81 (1.81, 4.37)	1.02 (0.57, 1.84)	1.03 (0.57, 1.88)
Normal^7^	63 (32)	2.0–3.6	Reference	Reference	Reference
Iron, μmol/L					
Lower than normal	92 (47)	<20	1.24 (0.87, 1.77)	1.02 (0.68, 1.53)	1.02 (0.68, 1.54)
Normal^8^	100 (51)	20–55	Reference	Reference (*n* = 168)	Reference (*n* = 168)
Elevated	4 (2)	>55	0.93 (0.22, 3.82)	0.91 (0.60, 1.37)	0.78 (0.18, 3.27)
sTfR, nmol/L					
Lower than normal	6 (4)	<10.6	0.87 (0.35, 2.16)	0.52 (0.2, 1.35)	0.52 (0.20, 1.35)
Normal^9^	123 (63)	10.6–29.9^9^	Reference	Reference	Reference
Elevated	67 (34)	>29.9	1.05 (0.73, 1.53)	0.91 (0.60, 1.38)	0.90 (0.58, 1.38)
Hemoglobin, g/L					
Anemic	111 (79)	—^10^	3.26 (1.75, 6.07)	2.75 (1.31, 5.76)	2.72 (1.29, 5.72)
Nonanemic	29 (21)	—^10^	Reference	Reference	Reference
ACT, g/L					
Lower than normal	2 (1)	<0.2	0.74 (0.10, 5.33)	0.95 (0.12, 7.38)	NA
Normal^11^	151 (77)	0.2–0.6	Reference	Reference	NA
Elevated	43 (22)	>0.6	1.89 (1.27, 2.80)	1.00 (0.60, 1.66)	NA

1 All assays were performed using plasma. Unadjusted model sample size, *n* = 196, except for hemoglobin models (*n* = 140); adjusted models, *n* = 168, except for hemoglobin models (*n* = 119) due to missing data. ACT, α_1_-antichymotrypsin; NA, not applicable; sTfR, soluble transferrin receptor.

2 There is no established clinical reference range for hepcidin, and distribution of raw transferrin data indicated tertile classification was informative and therefore both tertiles and clinical cutoffs were included for transferrin.

3 The category with the lowest risk of mortality served as the reference category.

4 Adjusted for HIV type (HIV-1 plus HIV-dual or HIV-2), age, gender, BMI, and absolute CD4 cell count (>500, 200–500, or <200 cells/μL) at HIV diagnosis. Gender was not included in regression models in which gender was used to establish clinical cutoffs (anemia, ferritin); age was not included when age was used to classify ferritin categories.

5 Adjusted for ACT (continuous) plus all variables in footnote 4.

6 Ferritin normal reference ranges: age 18–44 y: men, 12–200 μg/L; women, 12–150 μg/L; age ≥45 y: men, 12–300 μg/L; women, 12 to 200 μg/L (32).

7 Transferrin normal reference range: 2.0–3.6 g/L (33); no participants had above-normal transferrin concentrations.

8 Iron normal reference range: 20–55 μmol/L (33).

9 sTfR normal reference range: 10.6–29.9 nmol/L for living at low altitude and black (34).

10 Anemia: hemoglobin <13 g/L for men and <12 g/L for women (30).

11 ACT normal reference range: 0.2–0.6 g/L (33). A normal reference range was not provided by the assay manufacturer, and given assay method sensitivity and inconsistencies regarding the existence of a “normal” reference range for ACT, the normal limits should be interpreted with these considerations (28).

#### PCA of iron homeostasis and inflammation in HIV.

With the use of PCA (**Table 3**), 2 unique patterns were identified that accounted for considerable cumulative variance (68%) in a PCA model with all iron homeostasis variables (PCA1). The first pattern (PCA1.1) was greatly influenced by the trio of hepcidin-ferritin-transferrin, which together explained 43% of the total variance. The second pattern (PCA1.2) was primarily influenced by plasma iron-sTfR-hemoglobin, which explained 25% of the variance. Notably, the identified patterns were driven by nonoverlapping PCs (e.g., the observed variables did not contribute in a meaningful way to both patterns). In a second model (PCA2) that included all iron homeostasis variables plus ACT, similar grouping patterns were identified. In this model, which accounted for 66% of the variance, the first pattern (PCA2.1) explaining 45% of the variance was dominated by the quartet of hepcidin-ferritin-transferrin-ACT, and the second pattern (PCA2.2) explaining 21% of the variance was again primarily influenced by plasma iron-sTfR-hemoglobin. In both PCA models, the PC eigenvectors (e.g., the relative weighting attributed to each of the variables within a specified pattern) between hepcidin, ferritin, and transferrin with or without ACT were of comparative magnitude, whereas iron was comparatively more influential than sTfR or hemoglobin.

**TABLE 3:** PCAs of iron homeostasis and inflammatory variables in men and women at HIV diagnosis and Cox regression analyses of all-cause mortality^1^

					HR (95% CI)			
							Iron homeostasis index^5^	
PCA model	Grouping pattern identified by PCA	Total variance explained by pattern, %	PCs contributing to grouping pattern	PC eigenvector^2^	PC score,^3^ unadjusted	PC score,^3^ adjusted^4^	Unadjusted	Adjusted^4^
PCA1^6^	Pattern 1 (PCA1.1)	43.4	Hepcidin	0.54	1.72 (1.49, 1.99)	1.31 (1.07, 1.61)	1.37 (1.26, 1.50)	1.13 (1.00, 1.27)
			Ferritin	0.54				
			Transferrin	0.53				
	Pattern 2 (PCA1.2)	24.7	Iron	0.63	1.08 (0.93, 1.27)	1.00 (0.83, 1.20)	1.15 (1.03, 1.29)	1.08 (0.80, 1.45)
			sTfR	0.54				
			Hemoglobin	0.49				
PCA2^7^	Pattern 1 (PCA2.1)	44.8	Hepcidin	0.48	1.64 (1.44, 1.87)	1.26 (1.05, 1.51)	1.28 (1.19, 1.37)	1.08 (0.99, 1.89)
			Ferritin	0.48				
			Transferrin	0.46				
			ACT	0.45				
	Pattern 2 (PCA2.2)	21.3	Iron	0.61	1.06 (0.90, 1.24)	0.98 (0.82, 1.18)	1.15^8^ (1.03, 1.29)	1.08^8^ (0.80, 1.45)
			sTfR	0.56				
			Hemoglobin	0.46				

1 Unadjusted models sample size, *n* = 196; unadjusted with hemoglobin, *n* = 140; adjusted, *n* = 168, adjusted with hemoglobin, *n* = 119, due to missing data. ACT, α_1_-antichymotrypsin; PC, principal component; PCA, principal components analysis; sTfR, soluble transferrin receptor.

2 Ordered by relative loading (impact) of eigenvector where more important variables are assigned greater weights. Only meaningful (e.g., eigenvector >0.40) variables are presented.

3 PC scores represent a linear algebraic combination of all variables in the model and are weighted by eigenvectors.

4 Adjusted for HIV type (HIV-1/HIV-dual, HIV-2), age, gender, BMI, absolute CD4 cell count (>500, 200–500, or <200 cells/μL).

5 The iron homeostasis index represents the combined impact on mortality of PCA-identified patterns. Observed concentrations were classified into tertiles: a value of 1 was assigned to tertiles with the lowest HR (in Table 2) including the lowest hepcidin, ferritin, ACT, and sTfR tertiles and the highest transferrin, iron, and hemoglobin tertiles. Intermediate tertile = 2; otherwise = 3 (e.g., the greatest mortality HR in Table 2). Values were summed for individuals representing an overall iron homeostasis index, which was modeled as a single continuous variable.

6 Six-dimensional model including all iron homeostasis variables entered in tertiles.

7 Seven-dimensional model including all iron homeostasis variables plus ACT entered in tertiles.

8 The iron homeostasis index for PCA1.2 and PCA2.2 is equivalent because the same PC pattern (e.g., iron-sTfR-hemoglobin) was identified in both PCA models.

Mortality associations that used predicted PC scores, as well as an iron homeostasis index based on PCA-identified patterns, are presented in Table 3. PC scores derived from weightings assigned to variables in patterns PCA1.1 (hepcidin-ferritin-transferrin) or PCA2.2 (hepcidin-ferritin-transferrin-ACT) were significantly associated with mortality in unadjusted and adjusted analysis, whereas the PC scores derived from variables that grouped together in patterns PCA1.2 or PCA2.2 (both included iron-sTfR-hemoglobin) were not. Analysis of the iron homeostasis index showed that as the degree of iron deviation typified by the combination of elevated hepcidin, elevated ferritin, and lower transferrin (PCA1.1) or elevated hepcidin, elevated ferritin, lower transferrin, and elevated ACT (PCA2.1) increased, so did the mortality HR in unadjusted and adjusted models, although adjusted associations were attenuated. Like the PC scores modeling, the iron homeostasis index reflecting lower iron, higher sTfR, and lower hemoglobin was associated with increased likelihood of mortality. In both the PC score and iron homeostasis index models, the associations with hepcidin, ferritin, and transferrin, with or without ACT, were comparatively stronger than for iron, sTfR, and hemoglobin.

## Discussion

This study characterized hepcidin at the time of HIV diagnosis in relation to iron homeostasis, inflammation, and all-cause mortality in men and women with HIV infection. The findings show that hepcidin is integrally linked with the complex iron distribution that is associated with inflammation in HIV infection, a key cause of AI. This study also provides the first indication that elevated hepcidin is a risk factor associated with mortality in HIV infection by using unadjusted regression, although it is unclear whether the mechanism is mediated through its association with negative causes and/or consequences of inflammation, maladaptive iron distribution, or a reason yet to be identified.

**FIGURE 2:**
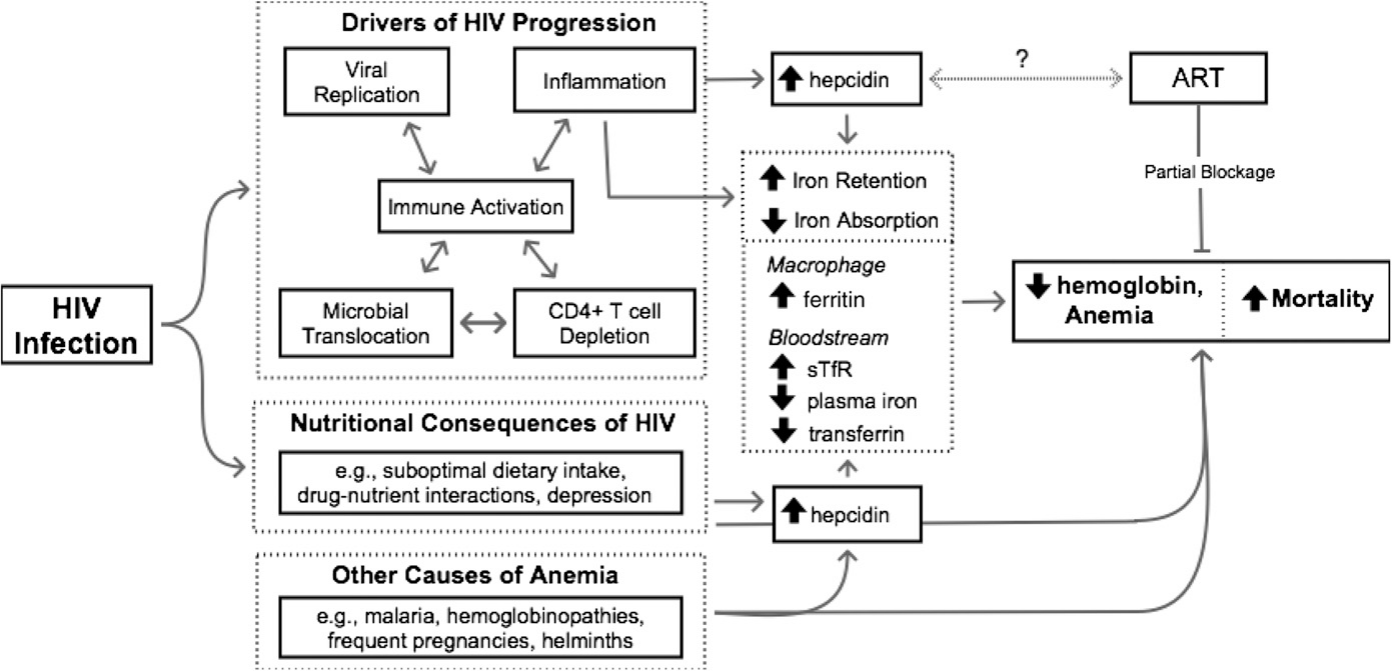
Pathways to HIV-associated anemia. A complex combination of interdependent factors including viral replication, microbial translocation, CD4 cell depletion, and chronic immune activation has been proposed as the driving force behind HIV disease progression (36). The production of proinflammatory cytokines (including IL-6) appears to both contribute to and be dependent on factors driving disease progression and, in turn, stimulates hepcidin production and triggers the acute phase response. As a result, there is an increase in hepcidin-mediated ferroportin degradation and a characteristic shift in iron from the bloodstream to the macrophage that is characterized by decreased plasma iron, decreased hemoglobin, decreased transferrin, increased sTfR, and increased ferritin, ultimately resulting in HIV-associated anemia. Although ART is known to resolve some (but not all) HIV-associated anemia, its role in the proposed pathway is currently unknown. Alternatively, HIV-associated anemia may be the result of nutritional consequences of HIV and/or other (non–HIV-related) causes of anemia. Arrows indicate connected entities that are part of a pathway whereby solid arrows represent established pathways and dotted arrows remain hypothetical; lines ending without arrows indicate inhibition or blockage. ART, antiretroviral therapy; sTfR, soluble transferrin receptor.

Although data regarding hepcidin in HIV infection remain limited, the findings from this study support those of an Indonesian study that reported that serum hepcidin was positively correlated with ferritin and inversely correlated with hemoglobin and absolute CD4 cell counts (15). Wisaksana et al. (15) also observed that elevated hepcidin at study entry was associated with an increased probability of starting tuberculosis treatment within 1–2 mo. Increases in macrophage iron stored in ferritin due to elevated hepcidin expression in an already immunocompromised host may alter the host-pathogen competition for iron and increase susceptibility to macrophage-tropic pathogens such as *Mycobacterium tuberculosis*. Although increased susceptibility to opportunistic infections such as tuberculosis may explain in part the poorer mortality prognosis associated with elevated hepcidin in the current study (35), the unavailability of cause-specific mortality data prevents testing this hypothesis with these data. Additional possible mechanisms linking elevated hepcidin and increased mortality are presented in **Figure 2**. The inverse association between hepcidin and absolute CD4 cells observed in this study and others (15) suggests that elevated hepcidin may be attributable to advanced disease stage and corresponding elevated levels of inflammation. Although increased hepcidin expression may be a consequence of inflammation, higher systemic hepcidin and the resulting transfer of iron from the bloodstream into macrophages may also contribute to HIV progression via enhanced HIV propagation and destruction of CD4 cells. In an in vitro study, iron export by ferroportin in the absence of hepcidin was associated with decreased HIV-1 transcription (14). Adding hepcidin counteracted the iron efflux, leading to increased intracellular iron and altered HIV production in CD4 cells and macrophages.

Hepcidin expression is regulated by inflammation (upregulation), erythropoiesis, and hypoxia (downregulation) and hepcidin concentrations are correlated with iron homeostasis biomarkers (e.g., ferritin, transferrin), although the relative hierarchy and biological importance of each under normal and clinical circumstances remain to be established (26, 37–39). By using statistical techniques designed to reduce redundancy of multiple correlated factors, further insight into the iron and infection relation is possible. In this study, PCA revealed 2 distinct patterns, each dominated by a different group of variables. Given the specific variables statistically assigned to each pattern, it is possible to conceptually speculate that the first pattern is associated with inflammation (hepcidin-ferritin-transferrin, with or without ACT) and the second with erythropoiesis (iron-sTfR-hemoglobin). Although both patterns may be influencing aspects of anemia and iron homeostasis, the regression analyses suggest that the inflammation-associated iron homeostasis pattern may be of greater relative importance in HIV. An understanding of the possible hierarchical nature of factors mediating hepcidin expression has not been elucidated for any infection. Early observations by Jonker et al. (38) suggest that they are complex. In a study in severely anemic Malawian children living in a region with high malaria and generalized infectious disease burdens, low hepcidin concentrations were observed. Because infection-induced inflammation was likely contributing to increased hepcidin expression, the very low hepcidin concentrations seem counterintuitive. The authors speculated that under conditions of severe anemia-associated hypoxia and resulting increased erythropoiesis, infection-associated inflammatory signals that usually upregulate hepcidin may be overridden or downregulated.

At this time, guidelines for anemia intervention in global settings include screening for malaria, treating helminth infection, and providing iron/folate supplements; however, self-treatment with the use of iron supplementation is common (40). This study provides support that elevated hepcidin is associated with anemia, and given the link with inflammation, screening to distinguish the type of anemia (e.g., AI and/or iron-deficiency anemia) would help to select the most appropriate anemia intervention. The evaluation of hemoglobin alone can determine neither the likely cause of anemia nor the best timing of interventions. Hepcidin concentrations have been shown to predict nonresponsiveness to oral iron therapy (41); and in combination with other iron biomarkers such as ferritin and/or transferrin, hepcidin may guide in whom and when iron-based anemia interventions may be ineffective (27, 42, 43). Many hepcidin interventions are in development, including hepcidin antagonists that mediate effects through inhibiting hepcidin production (e.g., anti-inflammatory agents such as anti–IL-6/IL-6R, anti–TNF-α), neutralizing hepcidin peptides (e.g., anti-calins, anti-hepcidin monoclonal antibodies), or interfering with hepcidin-ferroportin binding (e.g., thiol modifiers, anti-ferroportin monoclonal antibodies) (44, 45). Although lowering hepcidin may be of benefit for iron homeostasis related to anemia, it could also negatively alter the host-pathogen competition for iron (38). Iron supplementation during active infection may overcome the universal iron sequestration mechanism mediated by the innate immune response. If this occurs while simultaneously intervening to lower hepcidin concentrations, a flood of iron dumped into the periphery could negatively alter the host-pathogen competition for iron (46), making infection control a prerequisite to iron-based anemia interventions.

The role of ART in mitigating hepcidin and iron homeostasis mechanisms remains to be elucidated (Figure 2), and this study is limited by the use of biological archives collected before widespread ART usage. Although generalizability is limited to similar populations, the study findings have broad relevance because many people in developing countries present for HIV diagnosis with baseline characteristics similar to those in this study, others are diagnosed and lost to follow-up before ART is initiated, and some drop out of medical follow-up after ART initiation. For these groups, and as a reference for future studies seeking to identify and treat the subgroup of ART-treated individuals with residual or incident anemia (Supplemental Table 1), the current data are important. Medication usage (ongoing or newly initiated) around the time of individual plasma samples was unknown and should be considered for future studies. Although these findings point to the importance of hepcidin in HIV infection, a portion of the statistical inferences were based on data-derived tertile categorizations of hepcidin and further research to identify clinically relevant cutoff points is needed. Last, although elevated hepcidin was associated with mortality in unadjusted regression models, the association was not statistically significant in models adjusted for a number of known mortality covariates (e.g., CD4, BMI, gender, age). Further studies may help to clarify the independent hepcidin association.

In summary, hepcidin is a piece of the complex and dynamic relation that links HIV-associated anemia, iron homeostasis, inflammation, and mortality in HIV infection. Higher hepcidin concentrations at HIV diagnosis are associated with a greater likelihood of mortality in men and women, and an understanding of how hepcidin evolves and influences iron homeostasis throughout early and chronic HIV infection is needed. This is especially important because many people with HIV suffer from anemia before ART initiation, and because ART may not fully resolve inflammation or anemia. Overall, this study provides additional insight for the development of effective evidence-based decisions to prevent and manage HIV-associated anemia and maladaptive iron homeostasis occurring at all stages of HIV infection.

## Footnotes

9 Abbreviations used: ACT, α_1_-antichymotrypsin; AI, anemia of inflammation; ART, antiretroviral therapy; PC, principal component; PCA, principal components analysis; sTfR, soluble transferrin receptor.
